# Opinions and knowledge on globally important foodborne parasites among healthcare professionals at a tertiary teaching hospital in Nigeria

**DOI:** 10.1016/j.fawpar.2020.e00075

**Published:** 2020-02-21

**Authors:** Michael Akinwale Efunshile, Kingsley Onuoha Onwakpu, Lucy J. Robertson, Pikka Jokelainen

**Affiliations:** a Department of Medical Microbiology, Ebonyi State University, Abakaliki, Nigeria; b Department of Medical Microbiology, Alex Ekweme Federal University Teaching Hospital, Abakaliki, Nigeria; c Norwegian University of Life Sciences, Department of Food Safety and Infection Biology, Section for Microbiology, Immunology, and Parasitology, Parasitology Lab, Adamstuen Campus, Ullevålsveien 72, 0454 Oslo, Norway; d Laboratory of Parasitology, Department of Bacteria, Parasites & Fungi, Infectious Disease Preparedness, Statens Serum Institut, Artillerivej 5, 2300 Copenhagen S, Denmark

**Keywords:** Foodborne parasites, Knowledge gaps, Prevention, Survey

## Abstract

Foodborne diseases are important everywhere in the world, but the level of attention they receive varies by region. We surveyed the current opinions and knowledge regarding the globally most important foodborne parasites (FBP) among healthcare professionals in Abakaliki, Ebonyi State, Nigeria, by conducting a questionnaire survey among healthcare professionals in a tertiary hospital. We focused on the FBP ranked as the top five globally: *Taenia solium*, *Echinococcus granulosus*, *Echinococcus multilocularis*, *Toxoplasma gondii*, and *Cryptosporidium* spp., and gathered local expert opinions regarding their importance in Nigeria. Moreover, we surveyed the extent of healthcare community knowledge on transmission, manifestations and pathologies, and prevention of infections with these five FBP. Among the 205 healthcare professionals completing the survey, *T. solium* was selected as important in Nigeria by 143 (70%), *E. granulosus* by 91 (44%), *E. multilocularis* by 62 (30%), *T. gondii* by 132 (64%), and *Cryptosporidium* spp. by 123 (60%). Only 44 (21%) of the participants selected at least 15 of the 25 answers to knowledge questions that we considered as correct to select. The proportion selecting at least 15 of the correct answers was not statistically significantly associated with gender nor with experience level. Our results suggest that further education about FBP should target healthcare professionals at all levels.

## Highlights

• Knowledge on global top-5 foodborne parasites was evaluated at a Nigerian hospital.
• 21% of the respondents selected ≥15 of 25 correct answers to knowledge questions.
• Further education on foodborne parasites for healthcare professionals is needed.
• 70% of the respondents considered *Taenia solium* to be important in Nigeria.

## 1 Introduction

Foodborne diseases are public health problems that, in addition to impacting upon the lives of the individuals personally affected, can impair socioeconomic development at national and regional levels, and have potential for global spread due to increasing opportunities for travel and international trade (Robertson et al., 2014). Infectious agents of foodborne diseases include bacterial, viral, fungal and parasitic agents.

Many of the parasites causing foodborne diseases are neglected (Robertson, 2018). For many foodborne parasitoses there is a considerable period (ranging from days to decades) between infection and the appearance of symptoms, and this, along with other factors, means that source attribution for some parasites is challenging. In addition, an absence of pathognomonic manifestations and pathologies for some parasites and the lack of cheap and sensitive diagnostic techniques also likely further contribute to their neglected status. The long incubation period, the importance of the immunological status of the host, and the chronic sequelae many of these parasites may cause also make it difficult to estimate accurately the disease burden they cause – however it is clear that the burden is substantial (Torgerson et al., 2014; Kirk et al., 2015). Foodborne parasites (FBP) are particularly neglected in Sub-Saharan Africa where surveillance data are lacking, and their prevalence in the human population is largely unknown. This has been suggested to be due to lack of prioritization by relevant authorities or due to the importance of other pathogens, including parasites, such as *Plasmodium* spp. (FAO/WHO, 2014).

The United Nation's Food and Agriculture Organization (FAO) and World Health Organization (WHO) ranked foodborne parasites with the greatest global impacts, based on: 1) number of global illnesses, 2) global distribution, 3) mortality, 4) acute morbidity and chronic morbidity, and 5) trade and economic impacts (FAO/WHO, 2014). Parasites that made the top-five list were: *Taenia solium*, *Echinococcus granulosus*, *Echinococcus multilocularis*, *Toxoplasma gondii*, and *Cryptosporidium* spp. Infection with these foodborne parasites can present with a broad clinical spectrum: the outcomes range from mild symptoms (e.g., *T. gondii* infection) to potentially fatal diseases (e.g., echinococcosis, cerebral toxoplasmosis, *T. solium* neurocysticercosis, and severe cryptosporidiosis in the immunocompromised and/or pediatric population).

Although healthcare professionals are among the most important sources of knowledge and advice on FBP, their focus may be towards treating illnesses rather than preventing them. Furthermore, food-related health education of consumers nowadays often focuses on nutritional benefits and risks, rather than the potential for infection and how to avoid this. It has been concluded, already fifteen years ago, that physicians would benefit from targeted food-safety education to enhance their roles in educating patients (Wong et al., 2004).

We designed this study to survey the current knowledge on the globally most important FBP among healthcare professionals in a Nigerian tertiary teaching hospital. Although *E. multilocularis,* third among the top-5 parasites globally, has a Northern hemisphere distribution, as *E. granulosus* (sensu *lato*) is endemic in Nigeria (Ohiolei et al., 2019a, Ohiolei et al., 2019b), we would nevertheless expect knowledge of *E. multilocularis* to exist among health workers, especially that it would not be expected in Nigeria. The other four parasites among the global top-5 are all endemic in Nigeria. Thus, we mapped the opinions of healthcare professionals regarding the importance, in Nigeria, of the FBP considered to be in top five globally (FAO/WHO, 2014), and surveyed the extent of healthcare professional knowledge on transmission, manifestations and pathologies, and prevention of these parasitic infections. The results of this study may assist in identifying knowledge gaps that can be addressed by continuing medical education (CME). In addition, our results will also contribute to the body of available data from Africa to complement global surveillance of these parasites, as they continue to emerge and re-emerge globally from their neglected positions.

## 2 Materials and methods

### 2.1 Ethical considerations

Approval for this study was obtained from the ethical review board of the Alex Ekweme Federal University Teaching Hospital (AEFUTHA), Abakaliki, Nigeria. Participation was voluntary and anonymous. It was explained that by returning the questionnaire to the designated staff, the participants gave consent for their answers to be used for research purposes and to target continuous professional education. No personal data were collected.

### 2.2 Study design and setting

The study was a cross-sectional questionnaire study carried out among healthcare professionals at the clinical departments, including outpatient department, of AEFUTHA, Abakaliki, Ebonyi State, South East Nigeria, during September 2019. This specific hospital was used as the setting for this study due to accessibility to the Nigerian authors (MAE and KOO). Abakaliki is an agrarian community within the tropical rainforest zone.

### 2.3 Sample size and recruiting

Based of 95% confidence interval and a margin of error of 5%, and using a freely available online survey sample size calculator (http://fluidsurveys.com/university/survey-sample-size-calculator/), we determined that a sample of 218 respondents was sufficient for this study. To achieve this sample size, altogether 250 questionnaires were distributed to healthcare professionals, who were a convenience sample of the staff of AEFUTHA; attempts were made to ensure that all departments (including the outpatient department) were included by distribution of the questionnaire at the weekly departmental meetings. We aimed for a general overall view, and included all professions among healthcare professionals and had no limitation to their experience level. Participation was voluntary.

### 2.4 Survey instrument

A structured, pre-tested questionnaire (Supplementary file 1) was used to obtain information from participants. We collected socio-demographic data, opinions on importance of the five pre-selected FBP in Nigeria, as well as information on the participants' knowledge about transmission, manifestations and pathologies, and prevention of infections with these parasites. The questionnaire was in English, printed on paper, and two pages long. The parasites were referred to by their scientific names; these parasites do not have relevant local names in this region.

In the questionnaire, it was emphasized that the study aimed to survey the extent of healthcare community knowledge and to gather expert opinions, and that some questions did not necessarily have single correct answers. We also mentioned that the FBP included were those that had been ranked highest globally (FAO/WHO, 2014). Participants were asked to answer the questionnaire based on their current knowledge, without using any external information sources. It was possible for respondents to select none, one, or several responses to each question.

To the knowledge questions regarding the FBP (questions 2–5 in the questionnaire), we considered 25 answers as correct to select. These were chosen based on what we considered common knowledge on the parasites (e.g., the classical manifestations and pathologies the infections may cause). In the evaluation of the results, we focused on whether these answers were selected. Some other answers are also correct (e.g. less-known manifestations and pathologies of the specific parasite infections), but knowing them could be considered specialist knowledge, which was not the focus of this work.

We set selecting at least 15 (60%) of the 25 answers that were considered as correct to select as the cut-off to indicate selecting a high number of correct answers. This was based on a priori expected mean number of correct answers the respondents would select being 5–15 (20–60%), reflecting our experience from an earlier knowledge survey on *T. gondii*, where the mean number of correct answers to 17 knowledge questions was 7.5 (44%) (Efunshile et al., 2017).

### 2.5 Statistical analyses

The data obtained were entered into Excel spreadsheet. Descriptive statistics were carried out and associations between gender as well as experience level and selecting a high number of correct answers to knowledge questions were evaluated using Stata 13.1 software (Stata Corporation, TX, USA) and Open Epi software (www.openepi.com). We report *P*-values (Mid-P Exact, 2-tailed) for the comparisons.

## 3 Results

Forty-five (18%) individuals who received a questionnaire returned it unanswered and were omitted from the analyses. The final number of participants was thus 205. Of the 205 participants, 104 (51%) were female, 101 (49%) were male, and two were of other gender or did not answer this question. Different experience levels were represented: 59 (29%) had less than five years, 75 (37%) had five to ten years, and 71 (35%) had more than ten years of experience in the medical field. Among the participants, 60 (29%) were nurses, 33 (16%) were medical consultants, 71 (35%) were resident doctors, ten (5%) were laboratory scientists/scientific officers, and 31 (15%) had some other position.

Of the five parasites, *T. solium*, *T. gondii* and *Cryptosporidium* spp. were considered important in Nigeria by more than half of the participants. *Taenia solium* was marked as important by 143 (70%), *E. granulosus* by 91 (44%), *E. multilocularis* by 62 (30%), *T. gondii* by 132 (64%), and *Cryptosporidium* spp. by 123 (60%) of the participants.

The number of participants selecting each answer to the knowledge questions on transmission routes of the foodborne parasites to humans, typical manifestations and pathologies caused by the foodborne parasites, and practices that can help to prevent human infections with the foodborne parasites are shown in Table 1.

**Table 1:** The number and proportion of participants selecting each parasite as being relevant to the question or description in a questionnaire study among 205 health care professionals in a tertiary teaching hospital in Nigeria. The 25 answers that were considered correct to select are indicated by bold font and underlined.

	*Taenia solium*	*Echinococcus granulosus*	*Echinococcus multilocularis*	*Toxoplasma gondii*	*Cryptosporidium* spp.
**Transmission routes of the foodborne parasites to humans**					
Humans can become infected with this parasite by consuming undercooked meat of infected animals	**146 (71%)**	82 (40%)	73 (36%)	**71 (35%)**	44 (21%)
Humans can become infected with this parasite by consuming food/water contaminated with feces of infected hosts	**55 (27%)**	**63 (31%)**	**67 (33%)**	**106 (52%)**	**126 (61%)**

**Typical manifestations caused by the foodborne parasites in humans include**					
Diarrhea	108 (53%)	49 (24%)	35 (17%)	62 (30%)	**105 (51%)**
Hydrocephalus	20 (10%)	24 (12%)	24 (12%)	**66 (32%)**	39 (19%)
Epileptic seizures	**37 (18%)**	37 (18%)	35 (17%)	69 (34%)	54 (26%)
Cyst(s) in liver	58 (28%)	**84 (41%)**	**59 (29%)**	34 (17%)	22 (11%)
Ocular disease	25 (12%)	30 (15%)	59 (29%)	**47 (23%)**	18 (9%)

**Practices that can help to prevent human infections with the foodborne parasites**					
Good hand hygiene helps to prevent human infections	**108 (53%)**	**71 (35%)**	**66 (32%)**	**103 (50%)**	**111 (54%)**
Cooking meat thoroughly before eating helps to prevent human infections	**139 (68%)**	87 (42%)	80 (39%)	**86 (42%)**	47 (23%)
Washing vegetables before eating helps to prevent human infections	**85 (41%)**	**68 (33%)**	**63 (31%)**	**70 (34%)**	**87 (42%)**
Infections are vaccine-preventable in humans	8 (4%)	12 (6%)	9 (4%)	17 (8%)	19 (9%)

Of the 25 answers that we considered as correct to select (Table 1), the participants selected between none and 22; the mean was 10.2 and the median was ten. Altogether 44 (21%) selected at least 15 of the answers that we considered as correct to select. The proportion selecting at least 15 of the correct answers was not statistically significantly associated with male or female gender (20% in females and 24% in males; *P*-value 0.479), nor was it associated with a specific experience level (24% among those with <5 years of experience in the medical field and 21% among those with longer experience; P-value 0.614). Among the nurses, 13% selected at least 15 of the correct answers, while the proportion was 25% in the other groups (P-value 0.067).

## 4 Discussion

This is the first study to survey the extent of knowledge and to gather expert opinions about FBP among healthcare professionals in Nigeria. Focus on FBP in similar studies is rare (Xing-Da et al., 2019); similar mapping studies have tended to focus on bacterial pathogens (e.g., Angulo et al., 2008; Chehab et al., 2019).

The sample size obtained was slightly below the pre-calculated sample size. The target number was not achieved due to a surprisingly high number of participants not completing the survey form. It may be speculated that those who returned unanswered questionnaire were doubtful about the correct answers. However, we cannot be sure of this. Although a higher sample-size could have been useful in identifying patterns and differences, the data we obtained with our sample enables a baseline to be established. How well our sample represents the healthcare professionals in the hospital, and in other settings in the country, was not formally evaluated, but participation bias is likely. Participation was voluntary, and those with prior interest in the topic, as well as those with a positive attitude towards research, continuing medical education (CME), and improving the available knowledge, may be overrepresented. We should emphasize that regardless of the voluntary participation in this small study, the results only reflect knowledge at this specific location, and cannot be extrapolated to other hospitals in the region and in the country. It is possible that as a tertiary level teaching hospital, a higher level of knowledge might be expected, and it can be speculated that other hospitals at a lower level or with less emphasis on education may have less-knowledgeable staff.

One limitation of our study was that our approach for evaluating the characteristics of the non-respondents was limited. We did not evaluate the reasons for not participating nor for returning an unanswered questionnaire – we considered these individuals chose not to participate. The unused opportunity to investigate the non-respondents reinforces the need to emphasize that the sample was a convenience sample.

Of the five globally most important FBP, in the opinion of most respondents (>50%), *T. solium*, *T. gondii,* and *Cryptosporidium* spp., were important FBP in Nigeria. This is encouraging; the latter two are ubiquitous virtually globally, and *T. solium* occurs in most countries where pig-rearing systems allow access to non-controlled environments. It should be borne in mind, however, that as the respondents were informed in the questionnaire that FAO/WHO considered all these FBP important globally, they may have been influenced to also comment that they thought that they were important. This may have not only influenced the surprisingly high proportion of respondents marking *E. multilocularis* as important, but also the proportion indicating importance to the parasites that are known to be important in the country.

Indeed, a substantial proportion of participants (30%) marked *E. multilocularis* as being important in Nigeria; this was unexpected as this parasite is distributed in the northern hemisphere (Deplazes et al., 2017). However, it is possible that the respondents were confusing *E. multilocularis* with *E. granulosus* (which 44% marked as being of importance in Nigeria), as the latter parasite has been reported from a number of countries in Africa (Deplazes et al., 2017), including Nigeria (Adediran et al., 2014; Ohiolei et al., 2019a), although reports of human infection are sparse and the infection seems to be neglected (Ohiolei et al., 2019b). It should be noted that despite identification of cysts in the liver requires imaging techniques, *E. granulosus* was the parasite that most respondents selected as being associated with this manifestation (41%). A lower proportion associated *E. multilocularis* with this manifestation (29%), followed by even lower proportions of respondents associating this manifestation with the other three parasites (11–28%). Here, despite the expression ‘manifestation’ was perhaps not the optimal, it appears the respondents understood the meaning as intended (i.e. covering both clinical manifestations and pathologies).

There appeared to be room for improvement in knowledge about transmission routes of the FBP to humans. For example, only 35% of the participants indicated knowing that humans can become infected with *T. gondii* by consuming undercooked meat of infected animals. By contrast, in a previous questionnaire study, 69% of medical doctors indicated knowing that *T. gondii* infection can be meatborne (Efunshile et al., 2017). Also of concern is that only 27% of respondents considered that feces-contaminated fresh produce or water could be a transmission route for *T. solium*; as cysticercosis is a considerably more serious infection than taeniosis, and studies from Nigeria have shown a relatively high seroprevalence (e.g., 14.3% in Kaduna metropolis; Edia-Asuke et al., 2015), it is important that awareness is raised. A recent overview article on cysticercosis in West Africa, including Nigeria, emphasized the importance of targeted health education in order to tackle this disease (Weka et al., 2019); as long as healthcare professionals remain largely unaware of the transmission route this will be difficult.

The knowledge about typical clinical manifestations and pathologies is challenging to assess, but the results of our study may be useful in planning future studies and in targeting education. It needs to be emphasized that our selection of answers considered as correct was not complete, as many of the parasites can cause a wide variety of clinical manifestations and pathologies. For example, taeniosis has been associated with gastrointestinal signs, and cerebral toxoplasmosis can cause seizures. For this reason, we did not focus on identifying incorrect answers, and the selection of 25 answers that we considered correct to select was limited to include classical, typical manifestations that are known to be caused by these parasites. As less typical manifestations may not be known by all healthcare professionals, and pathologies can be very diverse, our emphasis was not on those respondents who selected manifestations or pathologies that may be less typical of the specific FBP but on those respondents who selected the classical manifestations or pathologies for a particular FBP (i.e. the 25 answers that were considered correct to select).

The results indicated some gaps in knowledge. For example, that *T. gondii* can cause ocular disease was known only by 23%. In our earlier questionnaire study, 37% of medical doctors answered that *T. gondii* infection can involve the eyes (Efunshile et al., 2017). Both proportions can be considered low. That only just over half of the respondents (51%) knew that cryptosporidiosis was associated with diarrhea is also of concern (indeed, more respondents (53%) associated *T. solium* infection with this symptom). Around 320 young children per 100,000 die of diarrhea in Nigeria annually, of which 14% are associated with *Cryptosporidium* infection, and a recent survey within this specific town, Abakaliki, indicated that of children under 5-years of age with acute watery diarrhea admitted to healthcare centers, over 5% had cryptosporidiosis, while typical treatment decisions focused on potential bacterial pathogens (Efunshile et al., 2018, Efunshile et al., 2019).

The answers about prevention options generally reflected the answers about transmission routes, indicating the participants selected their answers largely in a logical manner. Based on the percentages selecting each option, it appears that in this region, more focus could be placed on advocating washing vegetables before eating as a means of preventing foodborne parasitic infections. Mensah et al. (2012) provided some indications towards a holistic farm-to-fork approach for improving food safety in the African region; although FBP are barely mentioned within that article, emphasis is placed on preventing contamination, which is, indeed, crucial when considering fresh produce as potential vehicles for transmission of foodborne pathogens, including FBP.

What level of knowledge can be considered as “good” was not assessed in this survey, and was not the purpose of the study. The proportion selecting at least 15 of the answers that we considered correct to select did not differ statistically significantly according to the available background information variables. This suggests continuous professional education on FBP should target all healthcare professionals. Educating healthcare professionals on this topic might also have the potential to reduce the apparent underreporting of foodborne illnesses (Arendt et al., 2013; FAO/WHO, 2014).

Despite the limitations of our study, it provides a baseline overview on opinions and knowledge on FBP among healthcare professionals in a Nigerian hospital setting. The simple and straightforward study design is easily repeatable in other settings, including low-resource settings. We also hope that our study would inspire and encourage further education of health professionals on this subject. Further studies on barriers within food safety education – of healthcare professionals and by healthcare professionals – could be useful. Although we did not ask about it in this study, having interest to provide food safety information might be more common than providing it (Wong et al., 2004), which suggests unused potential. Surveying knowledge among students who are being educated to becoming healthcare professionals could allow optimal timing of targeted education (Xing-Da et al., 2019). Knowledge gaps can be filled, and the potential of healthcare professionals in educating the public about FBP, along with other foodborne pathogens, could be taken into use more efficiently. Previous surveys have investigated the levels of knowledge of food handlers regarding foodborne pathogens, which is, indeed, an important part of the workforce with respect to protection of consumers (Lee et al., 2017; El-Nemr et al., 2019). However, until healthcare professionals have solid knowledge on the transmission routes and prevention strategies, it is difficult to envisage the chain from food production to public health being adequately informed. The expertise and example of the healthcare professionals are needed to link food with human health.

The following is the supplementary data related to this article.

Supplementary file 1 Questionnaire used in the study on opinions and knowledge on globally important foodborne parasites among healthcare professionals at a tertiary teaching hospital, Nigeria, 2019.
