# Supplementary material for: Opinions and knowledge on globally important foodborne parasites among healthcare professionals at a tertiary teaching hospital in Nigeria
Source: Food Waterborne Parasitol. 2020 Feb 21;18:e00075. doi: 10.1016/j.fawpar.2020.e00075 (PMC7058820; doi:10.1016/j.fawpar.2020.e00075)
Supplement: Supplementary file 1 [file mmc1.pdf]

Foodborne parasites in Nigeria: opinions and knowledge among health workers

Efunshile Michael Akinwale, Igwe Daniel, Oyina Blessing, Robertson J. Lucy, Jokelainen Pikka

Dear colleague,

We invite you to participate in our **study on foodborne parasites in Nigeria**.
This study aims to survey the extent of our community knowledge and to gather expert opinions (please note that some questions do not have a "correct answer") on five foodborne parasite that have been ranked as the highest globally by FAO/WHO.

Please answer the questionnaire based on your current knowledge, without using any external information sources. You can select none, one, or several responses to each question.

Participation is voluntary and anonymous. By returning this questionnaire to our designated staff, you give your permission to use your answers for research purposes and to target continuous professional education.

1) BACKGROUND

Please circle all that apply:

A) Gender: (1) female, (2) male, (3) other, or no answer

B) Experience in medical field: (1) <5 years (2) 5-10 years (3) >10 years

C) Position: (1) Nurse, (2) Medical consultant,

(3) Resident doctor, (4) Laboratory Scientist,

(5) Other, please specify______________________

2) IMPORTANCE OF THE GLOBALLY TOP-RANKED FOODBORNE PARASITES IN NIGERIA

Please mark the parasites that you consider to be important in Nigeria with '**X**'

	Important in Nigeria
Taenia solium	
Echinococcus granulosus	
Echinococcus multilocularis	
Toxoplasma gondii	
Cryptosporidium spp.	

3) TRANSMISSION ROUTES OF THE FOODBORNE PARASITES

Please mark transmission routes to humans with '**X**'

	Humans can become infected with this parasite by consuming undercooked meat of infected animals	Humans can become infected with this parasite by consuming food/water contaminated with faeces of infected hosts
Taenia solium		
Echinococcus granulosus		
Echinococcus multilocularis		
Toxoplasma gondii		
Cryptosporidium spp.		

4) TYPICAL MANIFESTATIONS CAUSED BY THE FOODBORNE PARASITES INCLUDE

Please mark typical manifestations in humans with '**X**'

	Diarrhoea	Hydrocephalus	Epileptic seizures	Cyst(s) in liver	Ocular disease
Taenia solium					
Echinococcus granulosus					
Echinococcus multilocularis					
Toxoplasma gondii					
Cryptosporidium spp.					

5) PREVENTION OF INFECTIONS WITH THE FOODBORNE PARASITES

Please mark practices that can help to prevent human infections with '**X**'

	Good hand hygiene helps to prevent human infections	Cooking meat thoroughly before eating helps to prevent human infections	Washing vegetables before eating helps to prevent human infections	Infections are vaccine-preventable in humans
Taenia solium				
Echinococcus granulosus				
Echinococcus multilocularis				
Toxoplasma gondii				
Cryptosporidium spp.				

Thank you for your time!

Contact: Dr Efunshile, AM-drefunshile@yahoo.com, Ebonyi State University/Federal Teaching Hospital
